# Facile synthesis of ZnO nanobullets by solution plasma without chemical additives

**DOI:** 10.1039/d1ra05008g

**Published:** 2021-08-05

**Authors:** Kyusung Kim, Sangwoo Chae, Pil Gyu Choi, Toshio Itoh, Nagahiro Saito, Yoshitake Masuda

**Affiliations:** National Institute of Advanced Industrial Science and Technology (AIST) 2266-98 Anagahora, Shimoshidami, Moriyama Nagoya 463-8560 Japan masuda-y@aist.go.jp; Department of Chemical Systems Engineering, Graduate School of Engineering, Nagoya University Nagoya 464-8603 Japan

## Abstract

ZnO nano-bullets were synthesized using solution plasma from only Zn electrode in water without any chemical agents. In this sustainable synthesis system, the rapid quenching reaction at the interface between the plasma/liquid phases facilitates the fast formation of nano-sized materials. The coil-to-pin type electrode geometry, which overcomes the discharge interruption owing to the electrode gap broadening of the typical pin-to-pin type enables the synthesis of numerous nanomaterials through a stable discharge for 1 h. The as-prepared samples exhibited a high crystalline ZnO structure without post calcination, and the length and width were 71.8 and 29.1 nm, respectively. The main exposed facet of ZnO nano-bullets was the (100) crystal facet, but interestingly, the (101) facet was confirmed at the inclined surfaces in the edges. The (101) crystal facet has an asymmetric Zn and O atom arrangement, and it could result in a focused electron density area with relatively high reactivity. Therefore, ZnO nano-bullets are promising materials for applications in advanced technologies.

## Abstract 2

ZnO nano-bullets were synthesized using only Zn electrode and water by solution plasma and new electrode geometry improved discharge time up to 1 h.

## Graphical abstract

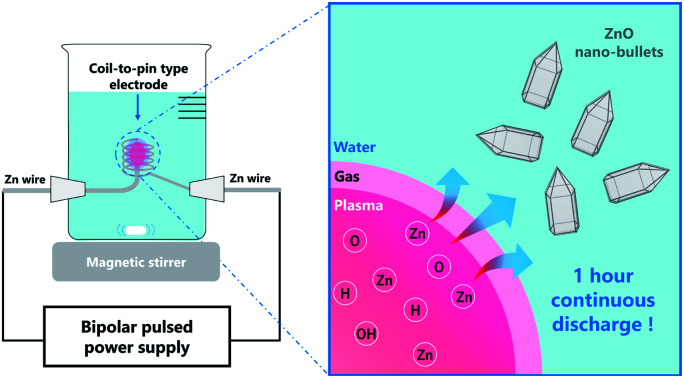

## 1. Introduction

Depending on the purpose, the synthesis of nanoscale metal oxide materials is innovative for wide applications such as gas sensors, catalysis, energy storage, conversion devices, and biomedical applications. Among the metal oxides, ZnO is used in numerous applications owing to its excellent chemical and thermal stability. Numerous synthesis methods such as hydro-thermal,^1,2^ sol–gel,^3^ solvothermal,^4^ and microwave,^5^ have been developed to fabricate ZnO nanomaterials. Recently, application of green chemistry has been increasing with a focus on the design of products and processes that minimize or eliminate the use and generation of hazardous substances.^6,7^ The eco-friendly method recommends the use of catalysts rather than stoichiometric reagents and avoids the generation of waste.^8–10^ Thus, numerous researchers have put their effort to find out more efficient and sustainable methods to synthesize nanomaterials. The plasma assistant synthesis method can replace the stoichiometric chemical synthesis method. The generation of electrons and radicals by plasma can lead to rapid chemical reactions without catalysts.^11–13^ In particular, the solution plasma method that directly discharges the liquid is a suitable synthesis method for manufacturing nanomaterials because of the radical reaction and rapid quenching at the plasma/liquid interface. In addition, discharge in an aqueous solution rather than in vacuum allows the facile preparation of metal oxides using oxidizing agents that are highly reactive such as O and OH radicals that originate from water.^14–16^ During the discharge in water, the water molecules are dissociated into H, O, and OH radicals, and these unstable species easily react with the neighboring species.^17,18^ Numerous researchers have investigated the formation of ZnO through these unique reactions using the solution plasma method.^19–22^ However, previous synthesis methods by solution plasma used chemicals such as ZnCl as the Zn precursor and K_2_CO_3_ as well as KCl as the electrolyte.^19–22^ In the case of ZnCl, the Zn precursor was used to generate Zn ions in the aqueous solution and Zn ions were reduced to Zn by electrons from the plasma. In the case of K_2_CO_3_ and KCl, the electrolyte must provide high conductivity to pure water to generate plasma on the surface of the electrode that is surrounding water. A gas layer as a dielectric barrier is formed by heating the electrode and the plasma is generated by breakdown through the air between the metal electrode and conductive water. These synthesis methods assisted by plasma could reduce the need for a chemical catalyst, but they still used chemical additives. Therefore, we developed a sustainable synthesis system using solution plasma that can reduce the use of chemical additives.

In our synthesis system, Zn electrode was used as the Zn source, and plasma was generated between the two metal electrodes through the water vapor channel. Therefore, Zn salt and other electrolyte chemical agents were not required for the formation of ZnO by plasma. Furthermore, long-term stable plasma could be generated by applying a coil-to-pin-type electrode geometry. In this study, we investigated the properties of ZnO nano-bullets synthesized *via* a sustainable synthesis method and explored their morphological advantages by analyzing the crystal structure of the crystal facet.

## 2. Experimental procedure

### 2.1 Preparation of ZnO nano-bullets

**Fig. 1:**
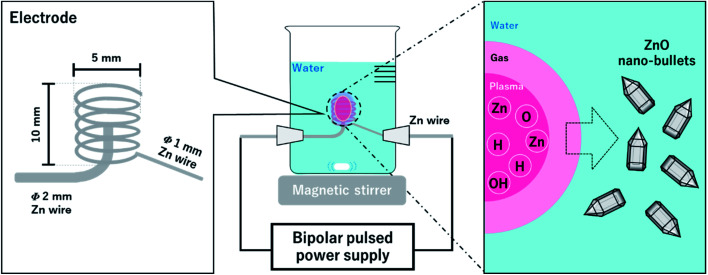
Scheme for the ZnO nano-bullet synthesis process by solution plasma.

A schematic experimental setup for synthesizing the ZnO nano-bullet without any chemical agent is shown in Fig. 1. Two Zn wires with different diameters were used as a pin (*Φ* = 2 mm) and coil (*Φ* = 1 mm) type electrodes. The inner diameter and length of the coil were 4 and 10 mm, respectively. Distilled water (500 mL) was used as the solvent. A bipolar pulse power supply (MPP04-A4-200, Pekuris) was used to generate the plasma between the pin and coil. The frequency and pulse width were controlled at 100 kHz and 1.0 μs, respectively, and the discharge inside the Zn coil was steadily conducted for 1 h in the open reactor while stirring the water at 200 rpm. After the reaction was complete, the white solution was separated by vacuum filtration. A polytetrafluoroethylene (PTFE) membrane filter with 0.1 μm of pore size (Millipore, Merck) was used in the filtration process. During discharge, optical emission spectroscopy (OES) was conducted to confirm the presence of reactive species in the plasma phase. OES measurement was performed in the wavelength range of 200 to 850 nm and an integration time of 100 ms was used.

### 2.2 Characterization

To confirm the reactive species in the plasma phase, the emitted light from the plasma was analyzed using an optical emission spectrometer (OES, USB4000-UV-vis, Ocean optics). The morphology of the samples was observed *via* field emission scanning electron microscopy (FE-SEM, JSM-6335FM, JEOL). Detailed structure was analyzed *via* transmission electron microscopy (TEM, JEM-2100F, JEOL) and high-resolution transmission electron microscopy (HR-TEM, JEM-2100F, JEOL). The crystal structure of ZnO nano-bullets was obtained on an X-ray diffractometer (XRD, SmartLab, Rigaku) with Cu Kα (*λ* = 1.5418 Å) radiation operating at 45 kV and 200 mA. The XRD patterns were acquired in the range of 20–100° with a step size of 0.01° and a scan speed of 1 min^−1^. The binding energy of the material was confirmed *via* X-ray photoelectron spectroscopy (XPS, PHI 5000 VersaProbe II, ULVAC) with monochromatic Al Kα radiation (*hν* = 1486.7 eV). A surface area analyzer (Nova 2200e, Quantachrome instrument) was used to obtain the nitrogen adsorption/desorption isotherms at 77 K cooled with liquid nitrogen. The specific surface area was determined using the Brunauer–Emmett–Teller (BET) method and the pore size distribution was calculated using the Barrett–Joyner–Halenda (BJH) method. The fluorescence spectrum of ZnO was obtained using a spectro-fluorometer (FP-6600, JASCO).

## 3. Result & discussion

**Fig. 2:**
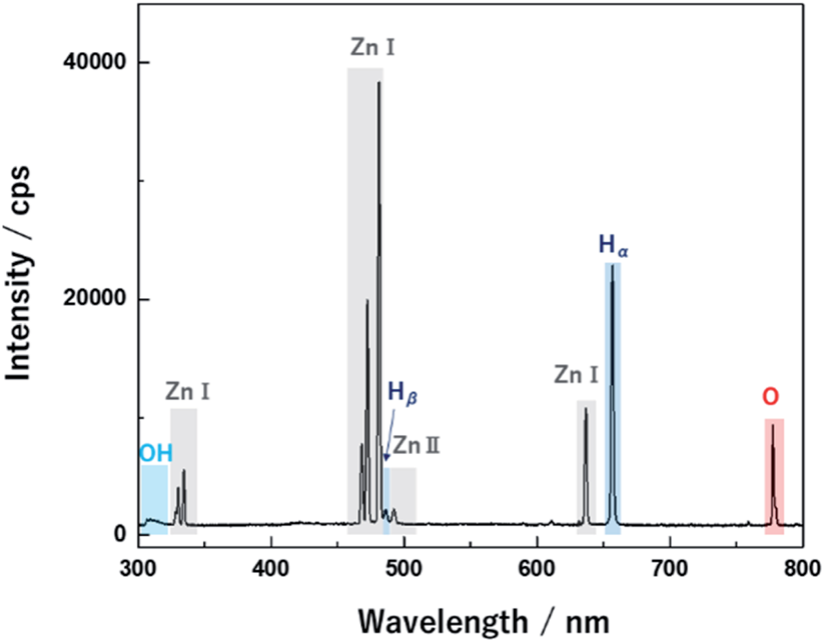
OES spectrum during discharge from the Zn electrode in the water medium.

Nano-bullet-structured ZnOs were synthesized using solution plasma. In the liquid phase, plasma can be generated through gas channels created by Joule heating of the electrode surface.^11^ During discharge, the reactive species were derived from the ionization of electrodes and the dissociation of water. The optical emission spectrum of the plasma was investigated to correlate the possible reaction process attributed to the reactive species. As shown in Fig. 2, Zn(i) (328, 330, 468, 472, 481, and 636 nm) and Zn(ii) (491 nm) emission peaks were obtained from the Zn electrodes. In addition, the peaks of H_α_ (656 nm), H_β_ (486 nm) O (777, 846 nm), and molecular OH (310 nm) radicals from the dissociation of water molecules were also observed.^23^

Therefore, the main chemical reactions that occur in the plasma could be suggested. The water molecules can be dissociated in the plasma phase as follows.

1 H_2_O → H˙ + OH˙

2 H_2_O → 2H˙ + O˙

Moreover, the presence of OH radicals in the plasma can promote oxidization through recombination to generate O radical as per the following reaction.

3 OH˙ + H˙ → O˙ + H_2_

Therefore, solution plasma provided an oxidizing atmosphere owing to the abundant generation of reactive oxidant species such as O and OH radicals. During the discharge, neutral Zn species came from the Zn electrode, and it was confirmed that Zn(i) peaks were dominant compared to the singly ionized Zn(ii). Therefore, neutral Zn generated in the plasma phase could form the ZnO cluster through a reaction with highly reactive O or OH radicals, in contrast to the typical chemical reaction initiated by the Zn ions.^24^

**Fig. 3:**
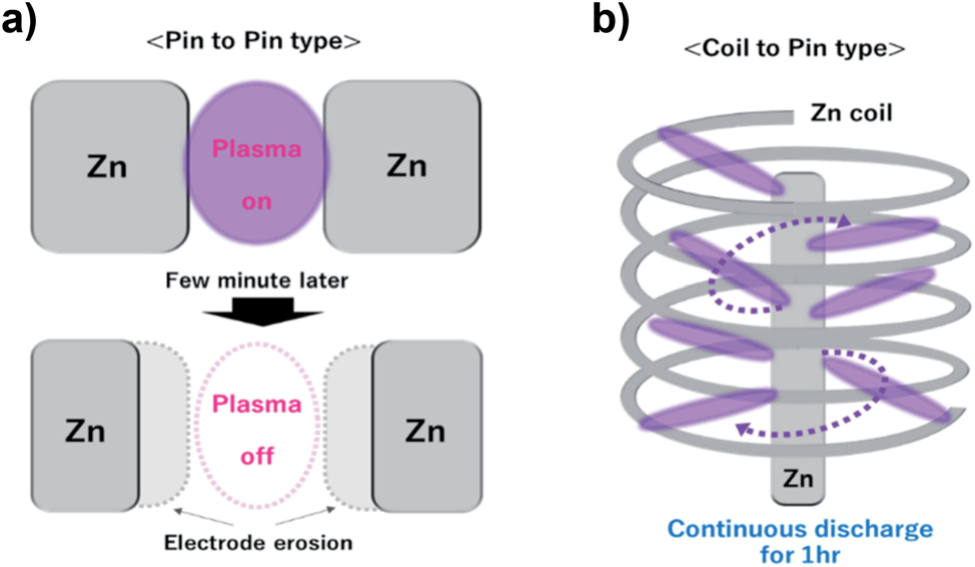
Scheme of electrode geometry: (a) pin-to-pin type and (b) coil-to-pin type.

In addition, the geometry of the electrode was modified to the coil-to-pin type (Fig. 3) for a long period of discharge without the *in situ* control of the electrode gap distance. Typically, the geometry of the pin-to-pin electrodes was used to synthesize nanomaterials in the solution plasma.^25,26^ It was difficult to maintain a long-term discharge during the fabrication of nanomaterials by sputtering the electrode material. This is because the gap between the electrodes gradually broadens owing to electrode erosion during discharge. In particular, the Zn electrode with a high sputtering rate (340 Å s^−1^) has the advantage of a rapid synthesis rate, but it also requires a modification for the stable discharge without broadening of the gap. Previously, mechanical equipment was utilized to maintain the gap between the electrodes to overcome gap broadening.^27^ However, our prepared coil-to-pin electrodes enhanced the discharge for a longer period without additional equipment.

**Fig. 4:**
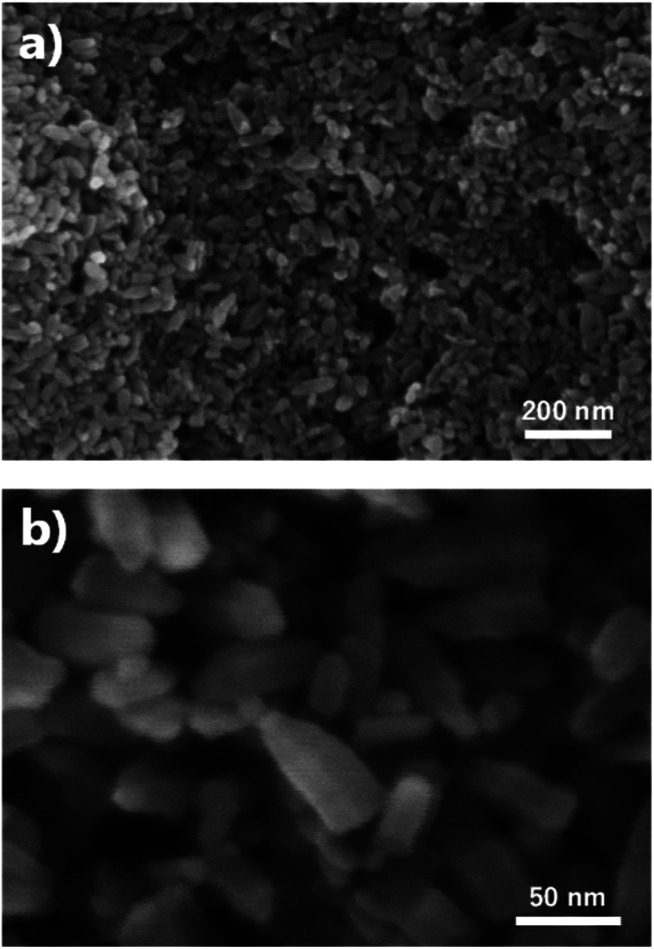
(a and b) Morphology of ZnO nano-bullet synthesized by solution plasma.

**Fig. 5:**
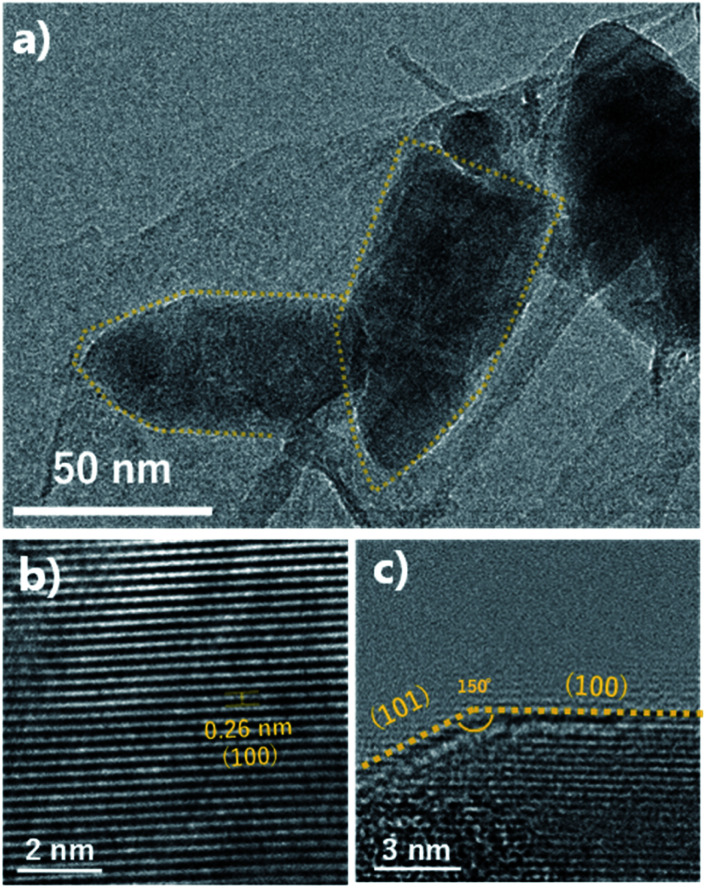
(a) TEM image and (b and c) HR-TEM image of ZnO nano-bullets synthesized by solution plasma.

The FE-SEM images show the morphologies of the ZnO nano-bullets. Uniformly synthesized ZnO nano-bullets with less than 100 nm were confirmed at low magnification (Fig. 4a). In addition, the bullet morphology of ZnO was observed in the high-magnification image (Fig. 4b). The mean length and width of ZnO nano-bullets were 71.8 and 29.1 nm, respectively. The results indicate that our plasma synthesis method uniformly fabricates nanoscale oxide materials. TEM and HR-TEM analyses were conducted to investigate ZnO nano-bullets. As shown in Fig. 5a, a bullet-shaped ZnO nanostructure is clearly observed. The lattice spacing in the HR-TEM image of a single ZnO nano-bullet with high crystallinity is presented in Fig. 5b. A layer distance of *d* = 0.26 nm, which corresponds to the *d*-spacing of the ZnO (100) planes, was observed. Fig. 5c shows an edge part of the ZnO nano-bullets, where the direction of growth was obliquely changed. The image exhibited the presence of the (101) crystal facet, which was inclined 150° from the (100) facet.

**Fig. 6:**
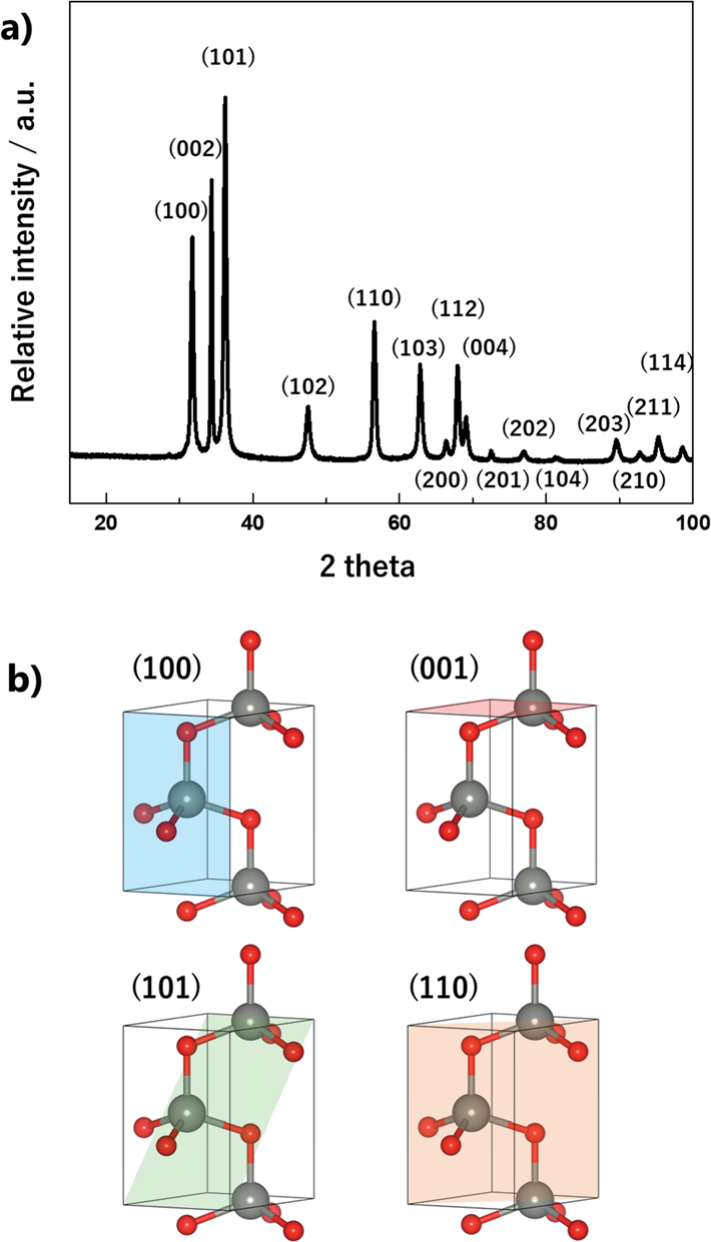
(a) XRD patterns of ZnO nano-bullets and (b) representative crystallographic planes of ZnO.

The crystalline structure of ZnO nano-bullets is shown in the Fig. 6a. All the diffraction peaks are indexed to a hexagonal wurtzite structure *P*6_3_*mc* (JCPDS no. 36-1451). Three dominant diffraction peaks associated with (100), (002), and (101) are observed at 31.7°, 34.3°, and 36.1°, respectively. Also, no additional peaks corresponding to Zn(OH)_2_ and pure Zn are observed. This result indicates an advantage of the solution plasma method: the post calcination process is not required to obtain only ZnO, even though the synthesis process is carried out at room temperature. In general, the post calcination process is necessary to improve the quality of a crystalline structure.^28^ In our case, oxygen radicals generated during the discharge may play a role in calcination.

**Fig. 7:**
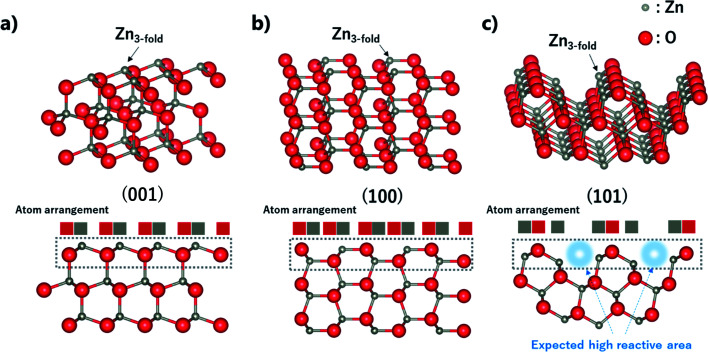
Crystallographic structure, side view, and atom arrangement of (002), (100), and (101) planes of Zn.

As mentioned, ZnO nanoparticles can be used in numerous applications, and considerable research has been conducted in the field of gas sensors and photocatalysts. The surface reaction of oxidation/reduction of molecules with the material is the main mechanism for the application. Thus, numerous studies have been performed to improve the surface reaction efficiency by economical modification without novel metal catalysts. The control of the exposed crystal facet is an effective method for increasing the reactivity of the surface. Although the material has the same composition, the total energy for the reaction differs depending on the type of crystal facet on the surface. This is because of the differences in the partial charge and electron density depending on the arrangement and spacing of the atoms. In addition, the coordinative saturation or unsaturation of the metal site is attributed to the difference in reactivity. The as-synthesized ZnO nano-bullets have a high potential for application in gas sensing and photocatalysis because the presence of the (101) crystal facet could increase the performance of ZnO. Fig. 7 shows the (001), (100), and (101) crystal facets of the O-terminated ZnO. Zn atom in all the crystal facets is bonded to three O atoms. Therefore, there is no difference in the reactivity of the Zn atom by coordination number. However, difference in the atomic arrangement at the surface layer was observed in the (101) crystal facet compared with the other facets. In the case of (001) and (100), the Zn and O atoms are alternately positioned in the horizontal direction. However, the (101) facet exhibits that two Zn atoms are arranged in succession, and this asymmetric arrangement of atoms could contribute to the high reactivity of ZnO. In practice, the effect of the (101) facet in enhancing the performance has been reported.^29,30^

**Fig. 8:**
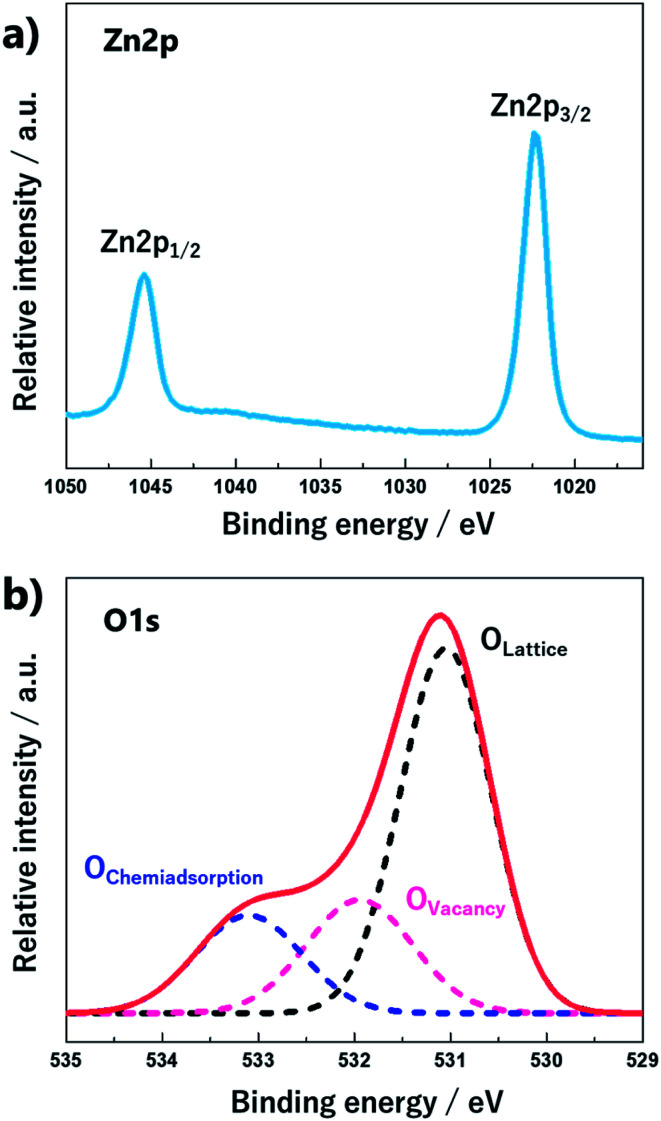
High-resolution XPS spectra of (a) Zn 2p and (b) O 1s for ZnO nano-bullet.

XPS spectra of the ZnO nano-bullets were deconvoluted to reveal the chemical bonding information. The high-resolution scan of the Zn 2p spectrum (Fig. 8a) with peaks at 1022.4 eV for 2p_3/2_ and 1045.4 eV for 2p_1/2_ indicated the oxidation state of Zn^2+^ in the form of ZnO in the sample. The energy difference between doublet binding energies was calculated to be 23 eV, which is consistent with the standard value of 22.97 eV. As shown in Fig. 8b, the O 1s spectra of ZnO nano-bullets were deconvoluted into three peaks by Gaussian distribution, located at 531.06, 531.94, and 533.95 eV. The peak at 531.06 eV was attributed to the O^2−^ ions in the wurtzite structure of the hexagonal Zn^2+^ ion array (O-lattice) and the peak at 531.94 eV was assigned to non-stoichiometric O. In addition, the peak located at 533.95 eV corresponds to the O chemisorbed state as dissociated oxygen.^31^

**Fig. 9:**
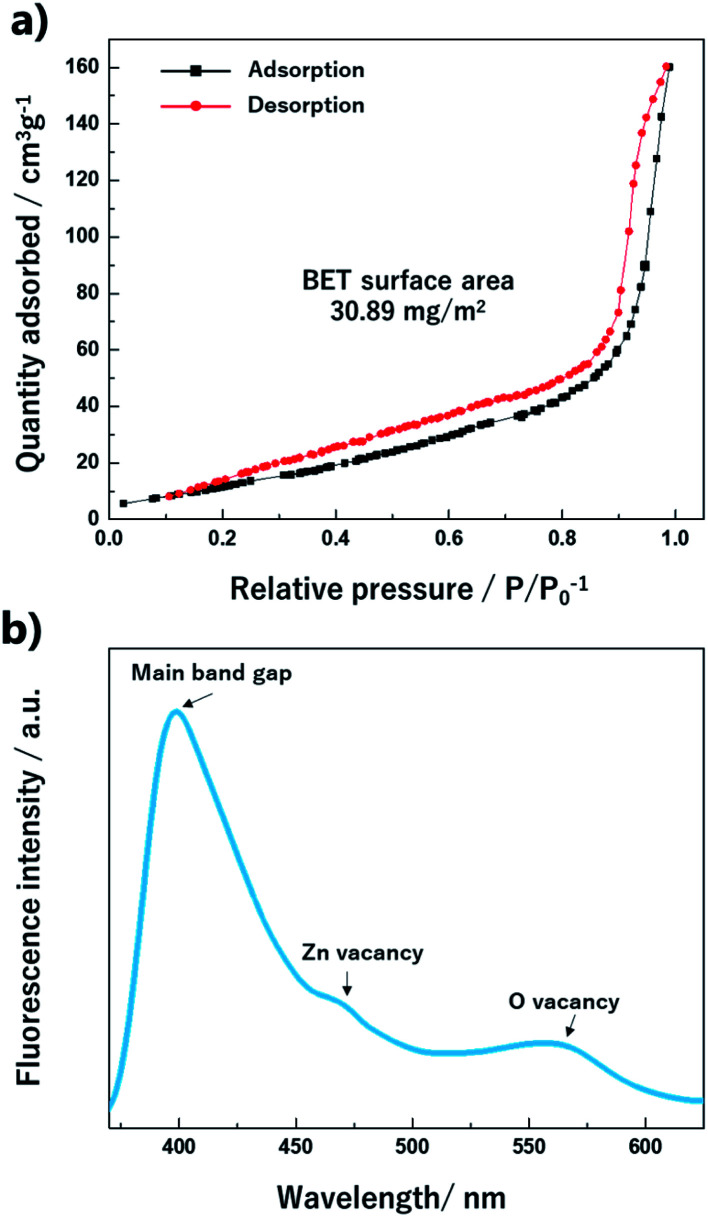
Isotherm curve with the (a) BET surface area and (b) fluorescence spectrum of the ZnO nano-bullet.

The specific surface area is a significant factor for the molecular reaction on the material surface. The surface area and porosity were characterized *via* the adsorption/desorption isotherm analysis of N_2_. In Fig. 9a, the isotherm curve shows a type-IV (a) behavior with a hysteresis loop.^32^ The calculated BET surface area for the ZnO nano-bullets was 30.89 m^2^ g^−1^. In the fluorescence spectrum (Fig. 9b) of the ZnO nano-bullet, the main peak was observed at 398 nm, which corresponds to the bandgap of ZnO. In addition, two small shoulder peaks attributed to vacancies were confirmed at 470 and 565 nm. The radiation from an electronic level of Zn vacancy emitted the fluorescence of 470 nm and an emission of 565 nm was generated when the energy level of the O vacancy was the acceptor.

## 4. Conclusions

In this study, ZnO nano-bullets were successfully synthesized using solution plasma in water without any chemical agent. In addition, the coil-to-pin electrode type allows stable discharge for 1 h, although a Zn electrode has a high erosion rate during the discharge in water. Uniform nano-sized ZnO materials were synthesized by rapid quenching at the plasma/liquid interface and post-calcination was not required for obtaining crystalline ZnO structures. The ZnO nano-bullet has a (101) crystal facet, which is a highly reactive surface owing to its asymmetric atomic arrangement. Thus, the as-synthesized ZnO nano-bullets could be applied in advanced technology owing to their advantages in size and morphology. Furthermore, solution plasma, which can perform the sustainable synthesis of nanomaterials, is a promising method for green chemistry. The demand for nanoscale metal oxide materials is constantly increasing for advanced technology applications. Thus, the development of sustainable synthesis methods should be considered in terms of environmental protection. Our plasma-assisted synthesis system in water is a simple and eco-friendly method. Moreover, various types of metal oxides and bimetallic oxides can be manufactured using other metal electrodes such as Ti, Cu, Fe, and Sn. Therefore, solution plasma has a high potential for application in green chemistry.

## Author contributions

Kyusung Kim: conceptualization, writing – original draft. Sangwoo Chae: formal analysis, data curation. Pil Gyu Choi: investigation, writing – review & editing. Toshio Itoh: visualization, resources, funding acquistion. Nagahiro Saito: methodology, validation. Yoshitake Masuda: supervision, funding acquistion.

## Conflicts of interest

There are no conflicts to declare.

## Supplementary Material
